# Axonal Transport: A Constrained System

**DOI:** 10.29245/2572.942X/2017/3.1118

**Published:** 2017-03-21

**Authors:** Clare C. Yu, Babu J. N. Reddy, Juliana C. Wortman, Steven P. Gross

**Affiliations:** 1 Department of Physics and Astronomy University of California, Irvine, Irvine, California, USA; 2 Department of Developmental and Cell Biology University of California, Irvine, Irvine, California, USA

**Keywords:** Pain, Transport, Theory, Neurons, Axons, Kinesin, Dynein, Molecular motors, Mitochondria

## Abstract

Long-distance intracellular axonal transport is predominantly microtubule-based, and its impairment is linked to neurodegeneration. Here we review recent theoretical and experimental evidence that suggest that near the axon boundaries (walls), the effective viscosity can become large enough to impede cargo transport in small (but not large) caliber axons. Theoretical work suggests that this opposition to motion increases rapidly as the cargo approaches the wall. However, having parallel microtubules close enough together to enable a cargo to simultaneously engage motors on more than one microtubule dramatically enhances motor activity, and thus decreases the effects due to such opposition. Experimental evidence supports this hypothesis: in small caliber axons, microtubule density is higher, increasing the probability of having parallel microtubules close enough that they can be used simultaneously by motors on a cargo. For transport toward the minus-end of microtubules, e.g., toward the cell body in an axon, a recently discovered force adaptation system can also contribute to overcoming such opposition to motion.

## Introduction

Eukaryotic cells are highly organized, and much of this organization is created and maintained by using active transport. Such transport utilizes a set of highways—the elongated polymers called microtubules (MTs)—combined with molecular motors—kinesin and dynein—that move along the highways. When such transport fails, the results can be catastrophic. Indeed, this is quite evident in neurons, where the extended nature of the cell puts particular demands on the transport system. Thus, impaired transport (via mutations in either the kinesin or dynein family of motors, or their regulators) is associated with neurodegenerative diseases^1,2^.

When considering potential impairment of transport, and its ultimate effects, one of the obvious questions that must be addressed is the challenge to such transport. That is, are there specific impediments to effective transport and are they cell-type specific? To date, only limited attention has been paid to potential differences between transport in the cell body (cytoplasm) and transport in the axon. However, a number of recent studies have started to address specifically this issue: that is, to determine whether there are differences in opposition to transport in these different cellular environments. One could imagine that varying opposition might arise from differences in boundary conditions (proximity to the cell walls), differences in the amount of polymerized actin, and even differences in the number of cargos moving in a particular area. In this review we summarize recent theoretical work suggesting that transport of larger cargos in neurons may face significant obstacles to motion. We then discuss experimental support for this theoretical suggestion. Finally, we suggest distinct complementary approaches for transport to overcome impediments.

## Transport near axonal boundaries likely feels significant opposition to motion

For the purposes of this review, we will confine our attention to transport in the axon where the microtubules are oriented with their plus ends away from the cell body^3,4^. (The microtubules in dendrites have a mixture of polarities, i.e., dendrites have both plus-end-out and minus-end-out microtubules.)

The geometry of the axon can present unique challenges to axonal transport absent in the cell body. The caliber or diameter of axons can range from less than 1 micron in small unmyelinated axons to about 10 microns in large myelinated axons. (In practice, electron micrograph images^5^ show a wide variation both in the caliber size and the longitudinal undulation.) The local number density of microtubules also has a wide range, from ~150 MTs/μm^2^ of cross sectional area of axoplasm in small caliber axons, to less than 15 MTs/μm^2^ in large caliber axons^6–9^. Cargo vesicles can vary in size from apparent diameters of 100 to 200 nm to greater than 600 nm in diameter in the squid giant axon (0.5 mm in diameter)^10,11^. The long cylindrical geometry of the axon—with the close presence of the axonal membrane—leads to two classes of effects, both of which impair transport. These are (a) a wall effect (reflecting a ‘no-slip’ boundary condition) in small caliber axons and (b) an enhancement of macromolecular crowding.

**Figure 1:**
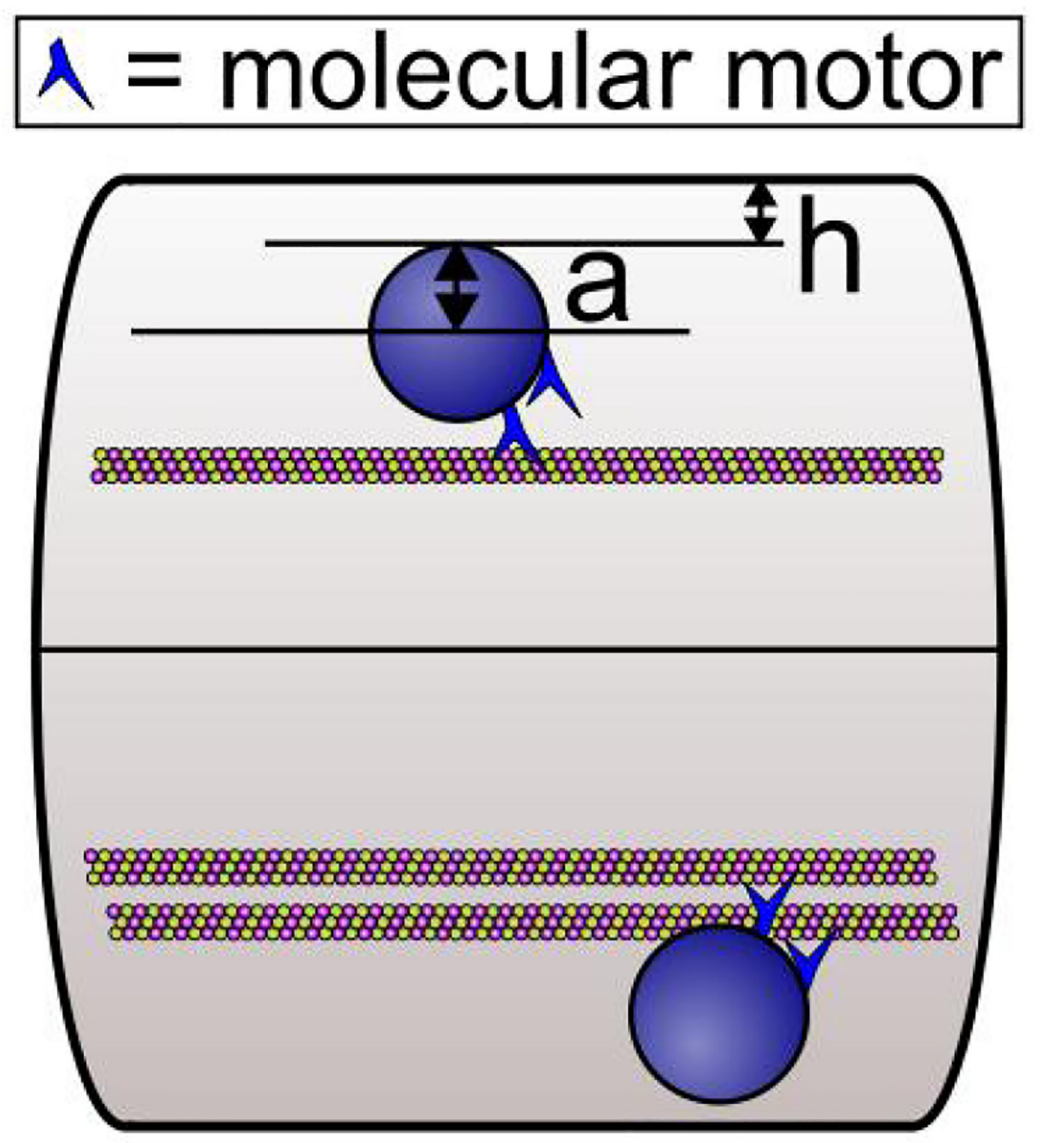
Cargos being hauled by motors along microtubules inside an axon. The spherical cargo has a radius of *a* in a cylindrical axon. The distance *h* is the closest approach of the cargo to the axon wall. The cargo has 2 motors. Two scenarios are shown. The top shows a single engaged motor hauling a cargo along a microtubule. The bottom shows both motors engaged in hauling the cargo. The key point of Wortman *et al*.^12^ is that the enhanced viscosity encountered by the cargo near the wall of the axon can be overcome by having multiple motors hauling the cargo along closely spaced parallel microtubules as shown at the bottom. Figure is from Wortman *et al*.^12^

The wall effect results from simple hydrodynamics: as a cargo moves along a microtubule in a small caliber axon as shown in Figure 1, it experiences a larger viscous drag than it would if it were moving in the cell body far from the cell walls. This is due to the no-slip or low-slip boundary condition of the cytosol at the axonal wall and also at the surface of the cargo, so that the closer the cargo is to the wall, the more shear there is, the larger the effective viscosity is, and the larger the opposition to motion. (The no-slip boundary condition refers to the fact that the fluid next to a surface cannot move or flow.) Calculations^12^ indicate that relatively close to the wall, the boundary effect is very large, and second, for cargos that are relatively large with respect to the caliber of the axon, e.g., filling it by half, the “wall” effect is evident even quite far away from the wall. For example, if we think of η_eff_ = Kη_∞_ as an effective viscosity, where η_∞_ is the viscosity in an infinitely large medium with no boundary and K a correction factor that accounts for the boundary, then η_eff_ can be 50 times that of water for K = 5 and η_∞_ = 10 times that of water^13^.

The second effect is an enhancement of the opposition to motion due to crowding, and conceptually results from an inability of large molecules to move out of the way of the cargo as it moves down the axon: as large molecules are pushed away from the oncoming cargo, their motion is impeded by the presence of the nearby walls or boundaries. These macromolecules include MT-associated proteins (MAPs) bound to MTs that have projections extending ~100 nm away from the surface of the MT^14,15^, neurofilaments whose C termini have long side arms that project 20 to 50 nm laterally outward from the filament core^15–17^, and plectins^18,19^. As the concentration of these macromolecules increases, the “base” viscosity of the medium also increases, further enhancing the wall effect^12^.

Using computer simulations, Wortman *et al*.^12^ demonstrated how the increased viscosity due to the wall effect and macromolecular crowding can impede axonal transport. They started by considering the simple case of a single motor hauling a cargo through an axon modeled as a long cylinder of uniform diameter with a microtubule centered along the axis of the axon. They found that even without any macromolecules, the run length decreased as the diameter of the axon decreased. For some parameter values, the effect of the increased drag because of the wall effect can be enough to halve the expected mean travel distance of a cargo that is hauled by a single motor. This would be more than enough to have significant physiological consequences^20^. Additional simulations showed that the amount of resistance to cargo motion depends on the size of the cargo relative to the axon diameter, on the cargo position relative to the axon wall, and on the extent that large macromolecules are in the locale^12^. In general, they found that the effective viscosity dramatically increased when *h*, the distance of the surface of the cargo to the inner wall of the axon, is comparable to or less than *a*, the radius of the cargo (see Fig. 1). Physically, small *h* meant that the cargo was close to the wall, and large *a* meant that there was a large amount of cargo surface area to enhance the viscous drag produced by proximity to the wall.

## Experimental confirmation of increased opposition to motion

Cultured primary CNS neurons from *Drosophila melanogaster* tend to have small caliber. Thus, they were chosen as an experimental system to test the hypothesis that larger cargos feel increased opposition to transport in axons. Since heavily loaded molecular motors move more slowly, and detach more readily from microtubules, the basic approach was to determine a) whether larger mitochondria had shorter travels and moved more slowly than smaller ones in neuronal processes (axons or dendrites), and b) whether these effects were either absent, or of decreased magnitude, in the cell bodies. When the motion of mitochondria was analyzed, it was found^21^ that in neuronal processes, larger mitochondria moved more slowly, and for shorter distances than did smaller mitochondria. Importantly, these size-dependent differences were absent in the cell body. Further, treating the processes with a hypotonic solution (causing them to swell, thus increasing their caliber) dramatically reduced size-dependent effects, and resulted in transport with velocities and run lengths quite similar to those found in the cell body.

Similar size-dependent velocities were reported for axonal transport of lysotracker-stained acidic organelles in rat DRG neurons^22^. In the squid giant axon (0.5 mm diameter), small vesicles, that typically had apparent diameters of 100 to 200 nm, had faster mean velocities (2.5 μm/s) than medium sized (200 to 600 nm) and large (greater than 600 nm) vesicles^10^. Also Yi *et al*.^20^ reported an increase in the number of arrested lysosomes/late endosomes larger than 1 micron in rat cortical neurons when the dynein adaptor LIS1 was inhibited with function blocking antibodies. It is unknown whether the cells adopt a different molecular mechanism in towing larger cargos. The above findings suggest that the opposition to motion described here is a generic effect in neuronal processes, valid for mammalian as well as *Drosophila* neurons, and for a variety of cargo types.

## Strategies to overcome resistance to axonal transport

The simplest way to overcome opposition to motion is to improve force production.

In principle, this can be done via three mechanisms: increasing the overall number of active motors to increase the number of engaged motors, changing geometry to promote increased engaged motors, or finally, enhancing the way the motors function together as a group. Studies from multiple groups^12,23,24^ indicate that the more motors that move a cargo, the further the cargo is expected to move and the greater the force that moves it. *In vivo*, multiple motors typically move a cargo^20,25–27^.

## Changing the overall number of active motors via CK2

It was recently reported^28^ that kinesin motors become inactive over time, that is, in a purified system, the number of active motors decreases as one waits. However, the inactivation is not permanent: it can be reversed by the protein Casein Kinase 2 (CK2)^29^, which re-activates the motors. While originally discovered *in vitro*, this process also occurs in cells: decreasing cellular CK2 levels was shown to decrease the force production of motors hauling a cellular lipid droplet^28^ (reflecting a decrease in the number of active motors moving the cargos, since such droplets are known to be moved by multiple motors). Thus, one strategy to increase the number of active motors moving a cargo is to increase CK2 levels, or promote its localization. While confirmed in a general cell context, the extent to which this occurs in a neuronal context remains to be explored.

## Control of microtubule geometry to affect motor engagement

Motor engagement can be increased substantially if there are multiple microtubules within reach of the motors on a cargo, e.g., doubling the number of microtubules that a motor can bind to effectively doubles the motor’s binding rate and the probability that a motor will be engaged in hauling the cargo. Wortman *et al*.^12^ used computer simulations to show that when several motors were present, the run length, i.e., travel distance, was considerably enhanced by having two microtubules available if the microtubules were within 100 nm of each other. Thus, their simulations suggested that to overcome the enhanced viscosity of the wall effect in small caliber axons, nearest-neighbor microtubules should be spaced within ~100 nm of each other so that both can be used to transport a given cargo.

Further, if an axon has microtubules randomly placed according to a Poisson distribution, they found^12^ that to have at least an 80% chance of having a pair of microtubules with a separation of 75 nm, the axon needs to have a microtubule density of ~100 microtubules/μm^2^. Thus the observation of 150 microtubules per μm^2^ of cross-sectional area of axoplasm for small unmyelinated axons^6–9^ means that small-caliber axons will have a high chance of having microtubules with a spacing less than 100 nm. In contrast, in large unmyelinated axons of ~10 μm in diameter, the microtubule density is less than 15 microtubules/μm^29^, implying a low probability that large caliber axons with randomly placed microtubules will have microtubules within 100 nm of one another. Thus, experimental microtubule densities are consistent with a hypothesis that for small caliber neurons, large cargos likely use multiple microtubules simultaneously to overcome opposition.

In principle, in addition to control of overall microtubule density, microtubule-microtubule spacing can be directly regulated via a variety of microtubule-associated proteins (MAPs) (see for example^30,31^). Overall control of both microtubule organization and axon caliber is also controlled by proteins such as ankyrin isoforms^32^. Finally, motors interact with the C-terminal tails of microtubules, and these interactions can affect how motors function^33^. By altering these C-terminal tails, post-translational modifications of microtubules can alter overall motor activity and function^34^.

## Enhancing group motor function under load: NudE and Lis1

A final way to improve cargo force production is to alter the way that the ensemble of motors on a cargo works together. Mechanistically, this has been described for cargos moved by cytoplasmic dynein toward the minus-end of a microtubule. (In an axon, this would be in the direction of the cell body. In an ordinary cell, this would be toward the nucleus.) When cargos experience load, and are stalled, the force production machinery can adapt by increasing utilization of the NudE and Lis1 cofactors^35,36^. This decreases the detachment rate of loaded motors, so that when under load, motors remain bound to the microtubules; such slowed detachment allows better additivity of multiple motor forces, and on average increases the average number of instantaneously engaged motors. Experiments^20^ suggest such adaptation is important for larger cargos in neurons, since decreasing Lis1 function results in an increase in stalled larger (but not smaller) neuronal lysosomal vesicles.

## Summary

To summarize, Wortman *et al*.^12^ hypothesized that axonal transport in small caliber axons can be hindered by the increased viscosity near the membrane; the existence of such opposition to motion was then experimentally confirmed^21^. This could be a factor in neurodegenerative diseases afflicting small fibers such as diabetes mellitus, Fabry’s disease, and chemotherapy-induced peripheral neuropathy^37^. For example, it is known that the amount of material conveyed via fast axonal transport is reduced by ~20% in the peripheral nerves of diabetic rats^38–40^. Ultimately, in healthy neurons, there appear to be multiple mechanisms to overcome such opposition, including increasing the number of active motors, increasing the number of locally available microtubules, and tuning single-motor properties to improve group function. The relative importance of these different mechanisms remains to be explored.
